# Supplementary material for: The BRCA2-MEILB2-BRME1 complex governs meiotic recombination and impairs the mitotic BRCA2-RAD51 function in cancer cells
Source: Nat Commun. 2020 Apr 28;11:2055. doi: 10.1038/s41467-020-15954-x (PMC7188823; doi:10.1038/s41467-020-15954-x)
Supplement: Supplementary file 1 — Supplementary Information [file 41467_2020_15954_MOESM1_ESM.pdf]

Supplementary information

The BRCA2-MEILB2-BRME1 complex governs meiotic recombination and impairs the mitotic BRCA2-RAD51 function in cancer cells

Zhang. J, et al.

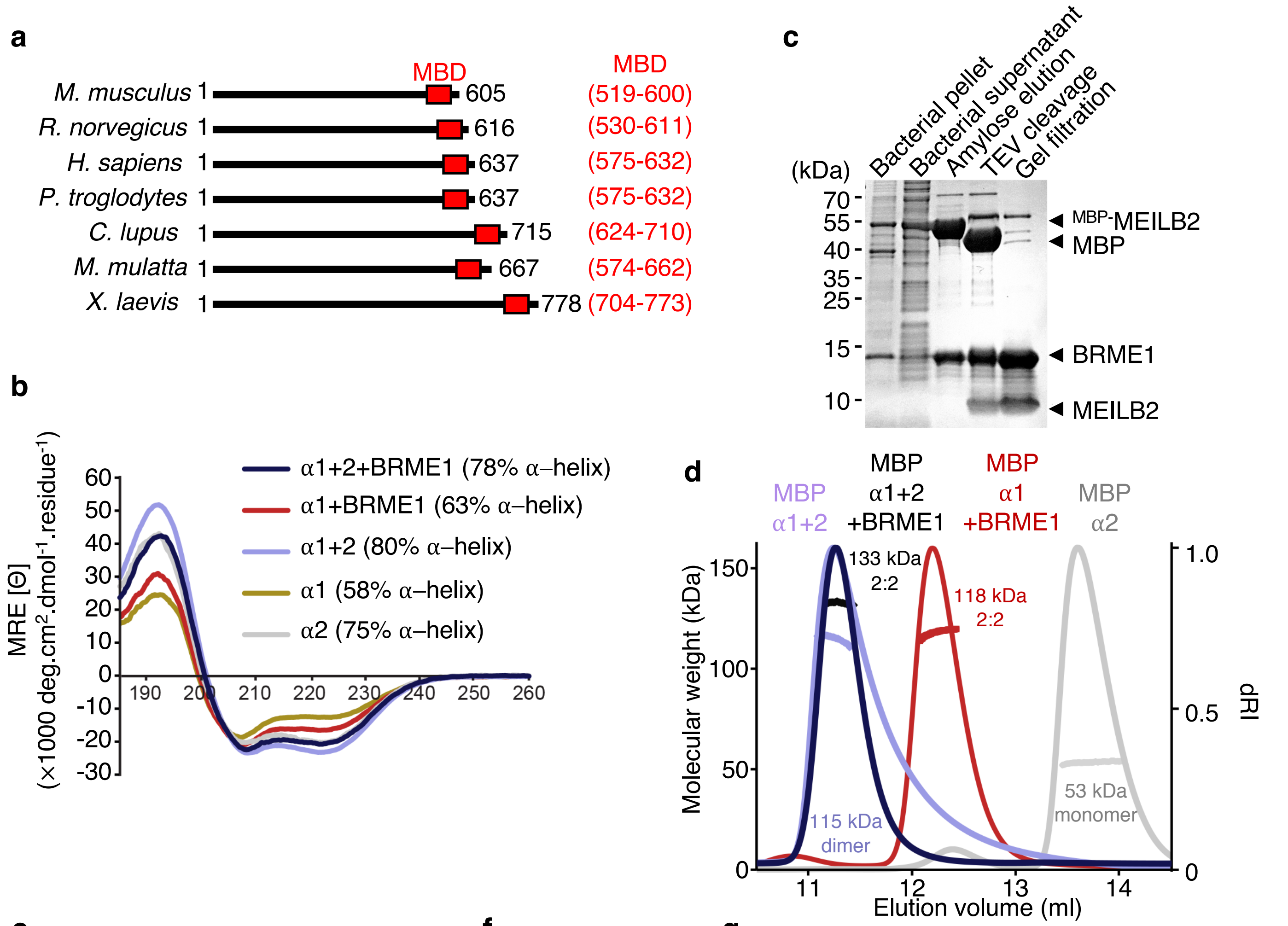

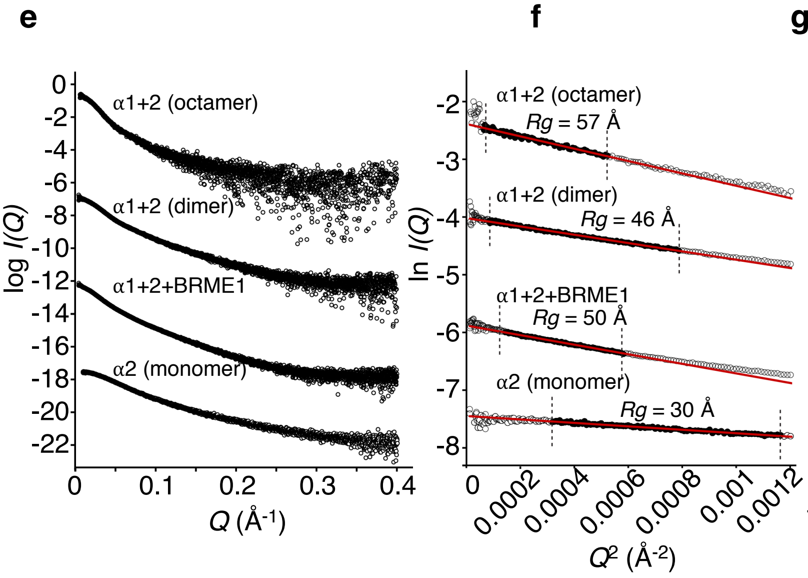

g

	MEILB2 α1+2 (18-122) +BRME1	MEILB2 α1+2 (18-122) dimer	MEILB2 α1+2 (18-122) octamer	MEILB2 α2 (51-122) monomer
**Guinier analysis**				
I(0) (cm^{-1})	0.095 $\pm 1.9\times10^{-4}$	0.041 $\pm 9.2\times10^{-5}$	0.022 $\pm 1.4\times10^{-4}$	0.027 $\pm 6.9\times10^{-5}$
Rg (Å)	50 ±0.74	46 ±3.9	57 ±0.50	30 ±0.19
q_{min} ($Å^{-1}$)	0.012	0.0091	0.0080	0.018
***P(r)* analysis**				
I(0) (cm^{-1})	0.097 $\pm 1.5\times10^{-4}$	0.041 $\pm 6.5\times10^{-5}$	0.022 $\pm 1.2\times10^{-4}$	0.027 $\pm 4.4\times10^{-5}$
Rg (Å)	53 ±0.11	48 ±0.086	59 ±0.38	30 ±0.084
D_{max} (Å)	190	160	200	110
Porod volume ($Å^3$)	104000	87800	368000	43400
MW from Porod volume (kDa)	61	52	217	26
V_C ($Å^2$)	569	521	885	212
MW from V_C (kDa)	53	48	112	12
DAMMIF ab initio modelling (30 models)				
Symmetry	P1	P1	P1	P1
NSD mean and sd	0.794 ±0.035	0.898 ±0.029	1.011 ±0.036	0.746 ±0.025
χ^2 (reference model)	1.23	1.31	0.973	1.05

Supplementary Fig. 1: Identification of BRME1

(a) BRME1 homologs. The *M. musculus* sequence was cloned in this study. Other sequences are from the NCBI protein database. *R. norvegicus* (XP_006255393.1), *H. sapiens* (NP_077299.3), *P. troglodytes* (XP_001171304.2), *C. lupus* (XP_005632861.1), *M. mulatta* (XP_001111192.2), *X. laevis* (XP_018109209.1). Rectangle: MBD region. (b) Far UV circular dichroism spectra with secondary structure composition estimated through deconvolution of the spectra. (c) SDS-PAGE of the co-purification of MEILB2α1+BRME1 complex. (d) SEC-MALS analysis. The differential refractive index (dRI) profiles are displayed with fitted molecular weights (Mw) plotted as diamonds across elution peaks. The MBP-fusion MEILB2α1+2+BRME1 (dark blue) and MEILB2α1+BRME1 (red) form 2:2 complexes of 133 kDa and 118 kDa (theoretical 2:2 – 138 kDa and 122 kDa), while MBP-fusions of MEILB2α1+2 (light blue) and α2 (grey) form dimers and monomers of 115 kDa and 53 kDa (theoretical – 115 kDa and 54 kDa), respectively. (e,f) SAXS analysis of α1+2+BRME1, α1+2 dimers and assemblies, and α2. (e) SAXS scattering data and (f) SAXS Guinier analysis to determine the radius of gyration (*Rg*), as shown, with linear fits in red and fitted data highlighted in black. The *Q.Rg* values were < 1.3. (g) Summary of SEC-SAXS data.

Fig. S1, Zhang

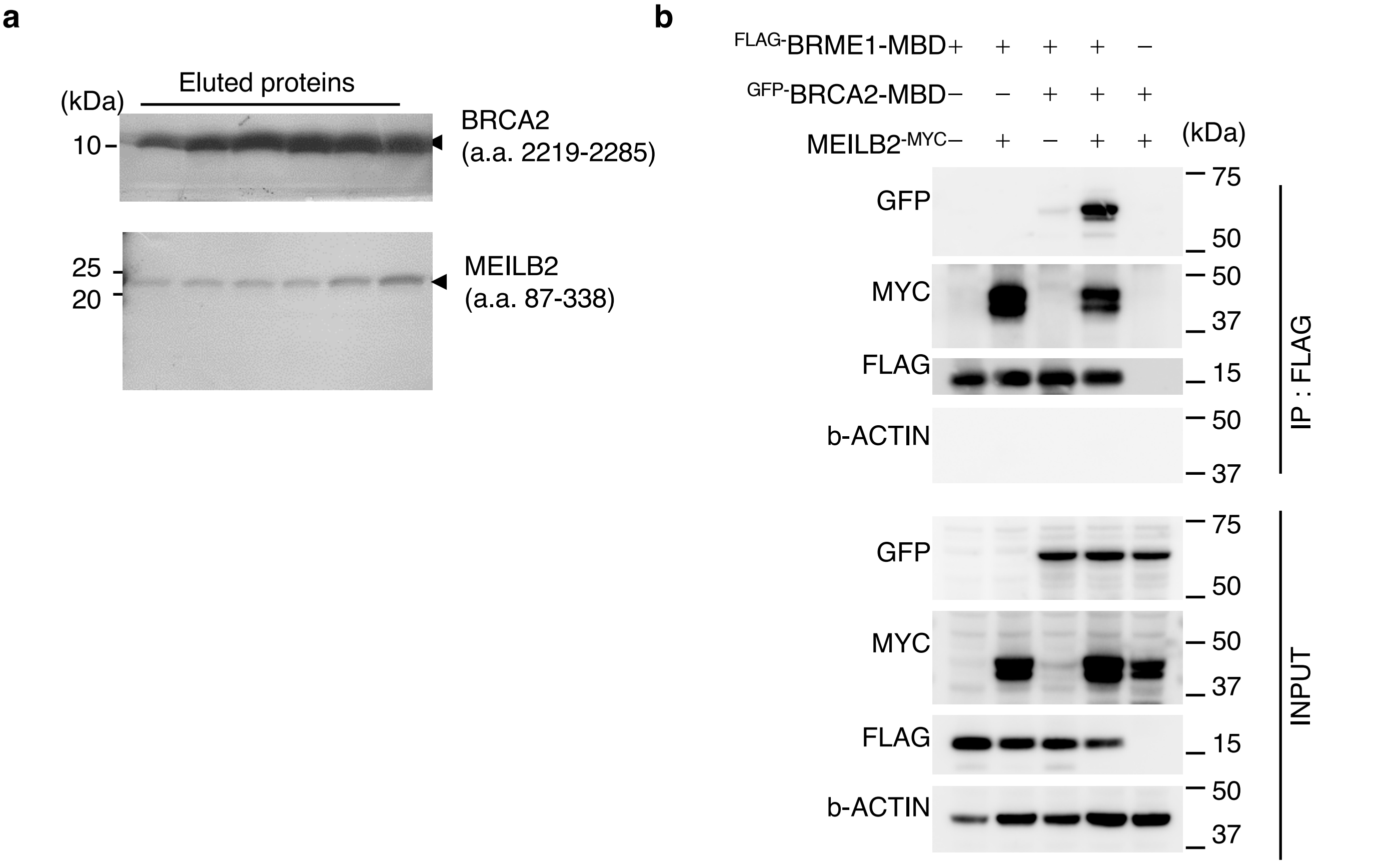

Supplementary Fig. 2: Characterization of BRCA2-MEILB2-BRME1 ternary complex
(a) Coomassie-stained gel showing the elution profiles of MEILB2 (a.a. 87–338, top) and BRCA2 (a.a. 2219–2285, bottom) from a cation exchange column. (b) Pulldown assay with the FLAG antibody from B16-F1 cells expressing FLAG-BRME1-MBD, MEILB2-MYC, and GFP-BRCA2-MBD (a.a. 2117–2339).

Fig. S2, Zhang

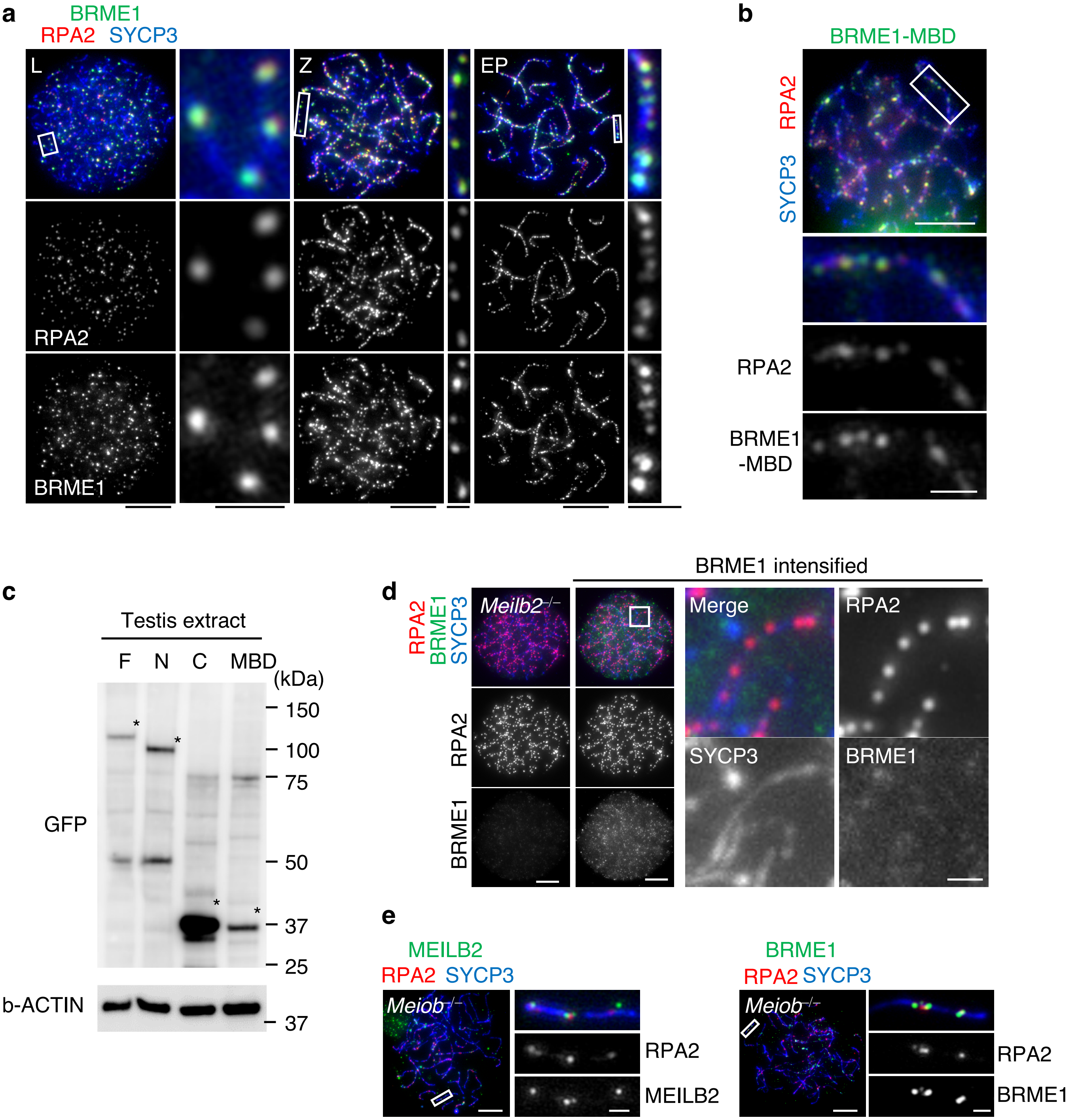

Supplementary Fig. 3: Characterization of BRME1 localization in WT spermatocytes
(a) WT spermatocytes stained with the indicated antibodies and DAPI. A total of 84%, 85%, and 84% of the BRME1 foci stained positive for RPA2, and 81%, 84%, and 80% of the RPA2 foci stained positive for BRME1 at the leptotene (L) (5 cells), zygotene (Z) (7 cells), and early-pachytene (EP) (5 cells) stages, respectively. Cells were pooled from two mice. (b) Immunostaining of zygotene spermatocytes expressing ^{GFP}-BRME1-MBD, showing the colocalization of RPA2 and GFP signals. (c) WB of testis extracts after electroporating BRME1 truncations, blotted with the indicated antibodies. Asterisks indicate the expected bands corresponding to each BRME1 truncation. (d) *Meilb2−/−* zygotene spermatocyte stained with the indicated antibodies and DAPI with intensified BRME1 signals. Note that axis-associated BRME1 foci are totally undetectable in *Meilb2−/−* spermatocytes. (e) *Meiob−/−* zygotene spermatocyte stained with the indicated antibodies and DAPI with intensified MEILB2 and BRME1 signals. Scale bars: 5 μm and 1 μm in the magnified panel. Source data are provided as a Source Data file.

Fig. S3, Zhang

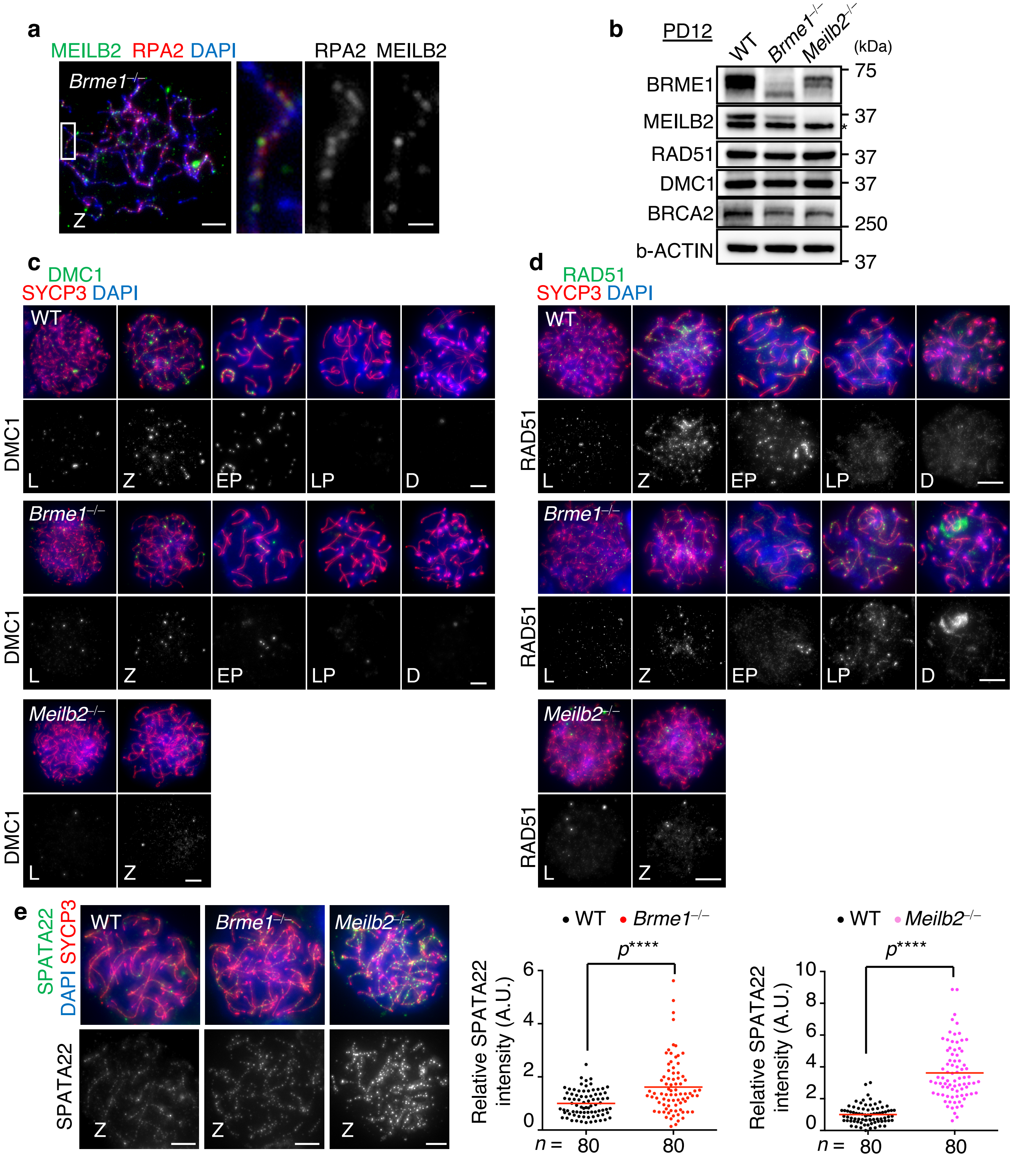

Supplementary Fig. 4: Reduction of recombinases and increase in SPATA22 intensity in *Brme1*−/− spermatocytes
(a) *Brme1*−/− zygotene spermatocytes stained with the indicated antibodies and DAPI with intensified MEILB2 signals. Note that axis-associated MEILB2 foci colocalized with RPA2. (b) WB of testis extracts from WT, *Brme1*−/−, and *Meilb2*−/− mice blotted with the indicated antibodies. Note that we used young mice at postnatal day (PD) 12 in order to equalize the cell population in each genotype. Asterisks indicate the non-specific bands detected by MEILB2 polyclonal antibody. (c,d) Spermatocytes from WT, *Brme1*−/−, and *Meilb2*−/− males stained with SYCP3 in red, DAPI in blue, and DMC1 (c) or RAD51 (d) in green. (e) Zygotene spermatocytes from WT, *Brme1*−/−, and *Meilb2*−/− males stained with the indicated antibodies and DAPI. The graph shows the relative signal intensity of SPATA22 foci normalized to the average values of WT. Red bars are the mean values, and n shows the analyzed foci number. Twenty randomly selected axis-associated foci were counted from each cell (four cells in total from a single mouse for each genotype). Knockout samples were prepared, stained, and quantified at the same time with WT control on the same glass slide. Leptotene (L), zygotene (Z), early- pachytene (EP), late-pachytene (LP), diplotene (D). All analyses used two-tailed t-tests. ****$p < 0.0001$. Scale bars: 5 µm and 1 µm in the magnified panel. Source data are provided as a Source Data file.

Fig. S4, Zhang

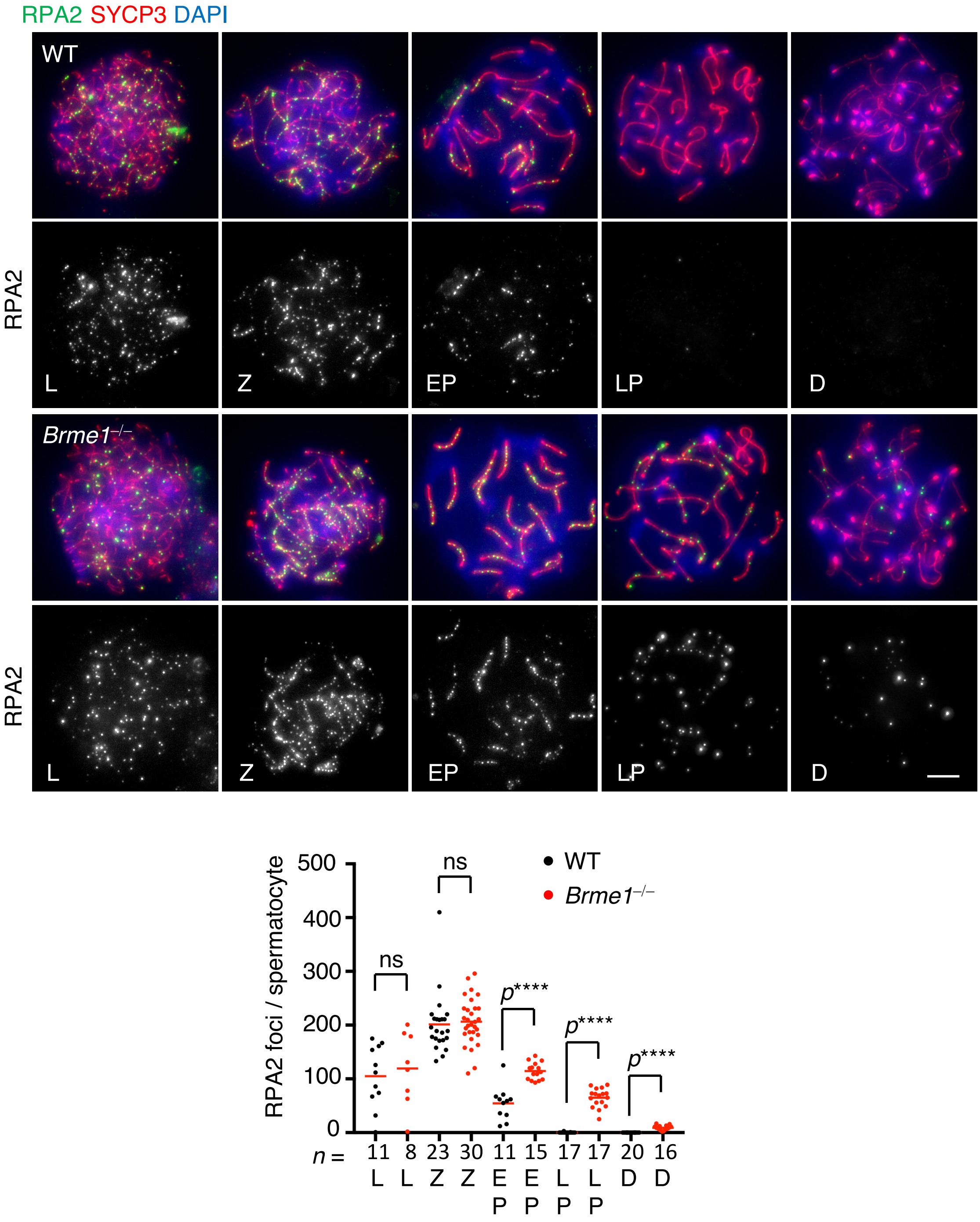

Supplementary Fig. 5: RPA2 foci persist until late prophase I in *Brme1*$^{-/-}$ spermatocytes

Spermatocytes from WT and *Brme1−/−* males stained with the indicated antibodies and DAPI. Leptotene (L), zygotene (Z), early-pachytene (EP), late-pachytene (LP), diplotene (D). Scale bars: 5 µm and 1 µm in the magnified panel. The graph is the number of RPA2 foci associated with the chromosome axes. Red bars are the mean values, and n shows the analyzed spermatocyte number pooled from three mice for each genotype. All analyses used two-tailed t-tests. ns: not significant. ****p < 0.0001. Source data are provided as a Source Data file.

Fig. S5, Zhang

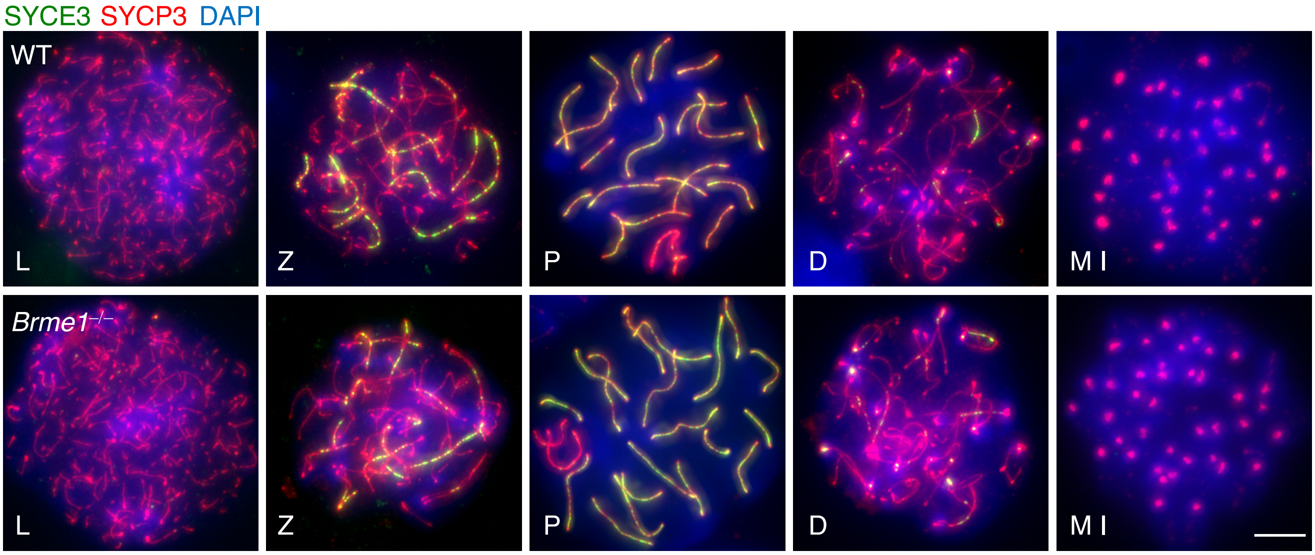

Supplementary Fig. 6: Progression of meiotic prophase I in *Brme1*$^{-/-}$ spermatocytes
Spermatocytes from WT and *Brme1*$^{-/-}$ males stained with the indicated antibodies and DAPI. Leptotene (L), zygotene (Z), pachytene (P), diplotene (D), and metaphase I (MI). Scale bar: 5 µm.

Fig. S6, Zhang

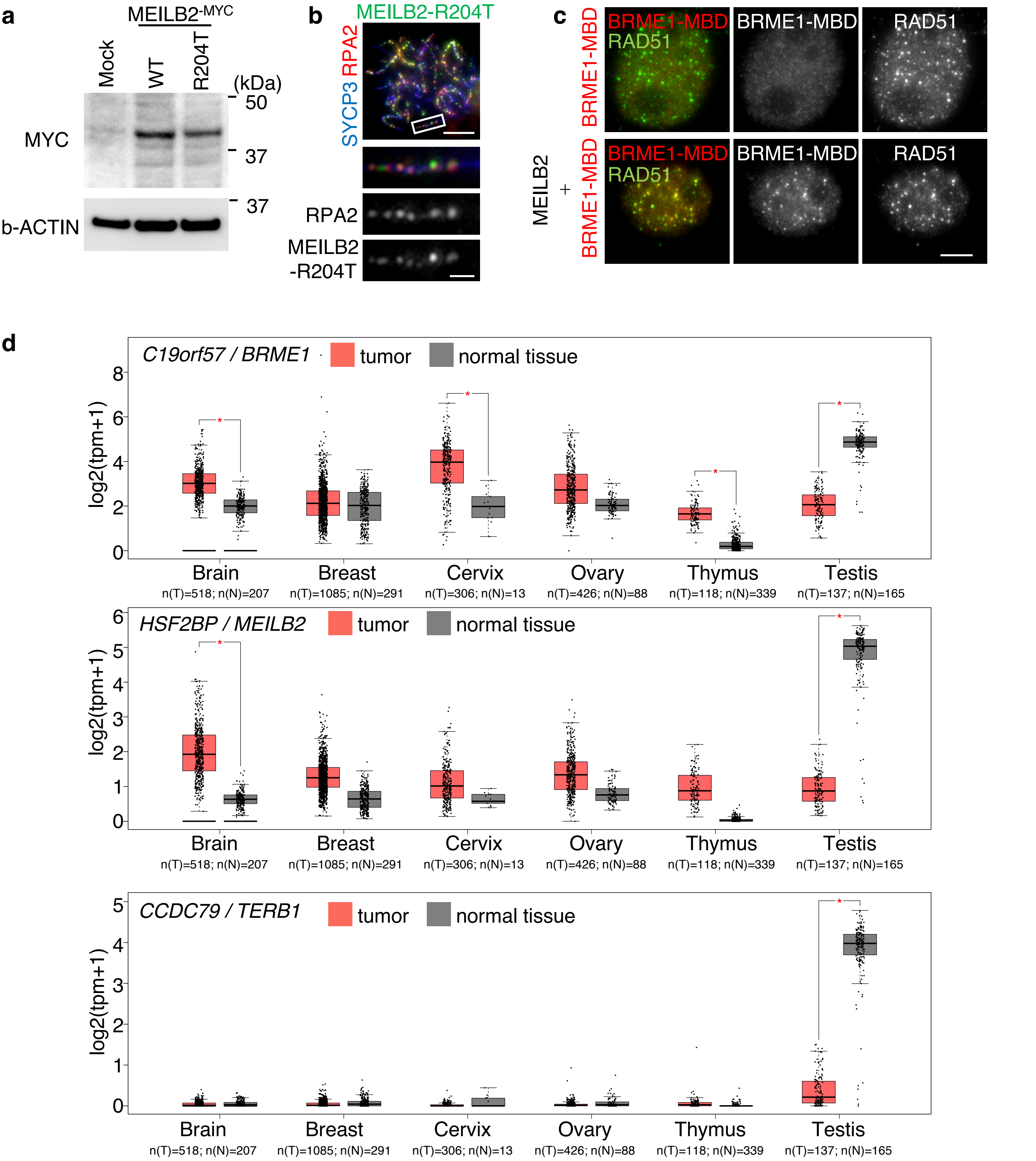

Supplementary Fig. 7: MEILB2 and BRME1 localization and expression in cancer cells

(a) Immunoblots of B16-F1 cell extracts after mock, MEILB2-MYC (WT), or MEILB2-R204T-MYC (R204T) transfections using the indicated antibodies. (b) Immunostaining of zygotene spermatocytes expressing GFP-MEILB2-R204T, showing the colocalization of RPA2 and GFP signals. (c) B16-F1 cells transfected with FLAG-BRME1-MBD (top) or both FLAG-BRME1-MBD and MEILB2-MYC (bottom) stained with RAD51 and MYC antibodies. (d) Expression of *C19orf57/BRME1* (top), *HSF2BP/MEILB2* (middle), and *CCDC79/TERB1* (bottom) in human tumor and normal tissue samples. The plot was generated from public RNA-seq data using the GEPIA server. The number of normal (n(N)) and tumor (n(T)) samples is indicated. Tpm, transcripts per kilobase million. Statistical significance was determined by one-way ANOVA. Note that both *BRME1* and *MEILB2* were upregulated in most of the cancer tissues, except for testicular cancer. The reduction of BRME1 and *MEILB2* expression in testicular cancer is likely due to the reduced numbers of meiotic germ cells in cancer tissues compared to normal tissues, as seen by the similar reduction in the expression of the meiotic gene *TERB1* in the testicular cancer samples. Scale bars: 5 μm and 1 μm in the magnified panel.

Fig. S7, Zhang
