# Supplementary material for: The BRCA2-MEILB2-BRME1 complex governs meiotic recombination and impairs the mitotic BRCA2-RAD51 function in cancer cells
Source: Nat Commun. 2020 Apr 28;11:2055. doi: 10.1038/s41467-020-15954-x (PMC7188823; doi:10.1038/s41467-020-15954-x)
Supplement: Supplementary file 3 — Reporting Summary [file 41467_2020_15954_MOESM3_ESM.pdf]

naturersearch

Corresponding author(s): Hiroki Shibuya

Last updated by author(s): Mar 24, 2020

# Reporting Summary

Nature Research wishes to improve the reproducibility of the work that we publish. This form provides structure for consistency and transparency in reporting. For further information on Nature Research policies, see Authors & Referees and the Editorial Policy Checklist.

## Statistics

For all statistical analyses, confirm that the following items are present in the figure legend, table legend, main text, or Methods section.

| n/a | Confirmed | |
|---|---|---|
| ☐ | ☒ | The exact sample size (*n*) for each experimental group/condition, given as a discrete number and unit of measurement |
| ☐ | ☒ | A statement on whether measurements were taken from distinct samples or whether the same sample was measured repeatedly |
| ☐ | ☒ | The statistical test(s) used AND whether they are one- or two-sided<br>*Only common tests should be described solely by name; describe more complex techniques in the Methods section.* |
| ☐ | ☒ | A description of all covariates tested |
| ☐ | ☒ | A description of any assumptions or corrections, such as tests of normality and adjustment for multiple comparisons |
| ☐ | ☒ | A full description of the statistical parameters including central tendency (e.g. means) or other basic estimates (e.g. regression coefficient) AND variation (e.g. standard deviation) or associated estimates of uncertainty (e.g. confidence intervals) |
| ☐ | ☒ | For null hypothesis testing, the test statistic (e.g. *F*, *t*, *r*) with confidence intervals, effect sizes, degrees of freedom and *P* value noted<br>*Give P values as exact values whenever suitable.* |
| ☐ | ☒ | For Bayesian analysis, information on the choice of priors and Markov chain Monte Carlo settings |
| ☐ | ☒ | For hierarchical and complex designs, identification of the appropriate level for tests and full reporting of outcomes |
| ☐ | ☒ | Estimates of effect sizes (e.g. Cohen's *d*, Pearson's *r*), indicating how they were calculated |

*Our web collection on statistics for biologists contains articles on many of the points above.*

## Software and code

Policy information about availability of computer code

| | |
|---|---|
| Data collection | no software was used for the data collection |
| Data analysis | Excel, GraphPad Prism 7, SoftWoRx, Proteome Discoverer version 2.2, Mascot 2.5.1, GEPIA server, ProtParam, ASTRA 6, CDSSTR algorithm, ScÅtter 3, PyMOL Molecular Graphics System, DAMMIF, PRIMUS |

For manuscripts utilizing custom algorithms or software that are central to the research but not yet described in published literature, software must be made available to editors/reviewers. We strongly encourage code deposition in a community repository (e.g. GitHub). See the Nature Research guidelines for submitting code & software for further information.

## Data

Policy information about availability of data

All manuscripts must include a data availability statement. This statement should provide the following information, where applicable:

- Accession codes, unique identifiers, or web links for publicly available datasets
- A list of figures that have associated raw data
- A description of any restrictions on data availability

The data supporting the findings of this study are available from the corresponding author upon reasonable request.
Requests for further information and requests for resources and reagents should be directed to Hiroki Shibuya (hiroki.shibuya@gu.se).

# Field-specific reporting

Please select the one below that is the best fit for your research. If you are not sure, read the appropriate sections before making your selection.

☒ Life sciences ☐ Behavioural & social sciences ☐ Ecological, evolutionary & environmental sciences

For a reference copy of the document with all sections, see nature.com/documents/nr-reporting-summary-flat.pdf

# Life sciences study design

All studies must disclose on these points even when the disclosure is negative.

| | |
|---|---|
| Sample size | No statistical method was used to predetermine sample size. We followed the conventional way of quantification accepted in many of the published paper in meiosis research field and determined the sample size. |
| Data exclusions | No data was excluded. |
| Replication | Each conclusion in the manuscript was based on results that were reproduced in at least two independent experiments and in at least two independent mice of each genotype. |
| Randomization | Mice were categorized based on their genotypes. The genotypes were determined by PCR. |
| Blinding | The investigators were not blinded to allocation during the experiments or to outcome assessment.<br>This is because the phenotypes were quite obvious that observer can be sure without blind test.<br>Further, the observer unbiasedly and carefully performed the quantification with enough sample number to make sure the conclusion. |

# Reporting for specific materials, systems and methods

We require information from authors about some types of materials, experimental systems and methods used in many studies. Here, indicate whether each material, system or method listed is relevant to your study. If you are not sure if a list item applies to your research, read the appropriate section before selecting a response.

## Materials & experimental systems

| n/a | Involved in the study | |
|---|---|---|
| ☐ | ☒ | Antibodies |
| ☐ | ☒ | Eukaryotic cell lines |
| ☒ | ☐ | Palaeontology |
| ☐ | ☒ | Animals and other organisms |
| ☒ | ☐ | Human research participants |
| ☒ | ☐ | Clinical data |

## Methods

| n/a | Involved in the study | |
|---|---|---|
| ☒ | ☐ | ChIP-seq |
| ☒ | ☐ | Flow cytometry |
| ☒ | ☐ | MRI-based neuroimaging |

## Antibodies

| | |
|---|---|
| Antibodies used | The following antibodies were used: rabbit antibodies against BRME1 (this study), BRCA2 (this study), MEILB2 7, GFP (Invitrogen; A11122), DMC1 (Santa Cruz Biotechnology; sc-22768), RAD51 (Thermo Fisher Scientific; PA5-27195), SPATA22 (Proteintech Group Inc; 16989-1-AP), SYCE3 (Shibuya lab), and MEIOB (EMD Millipore; ABE1414); mouse antibodies against BRME1 (this study), DMC1 (Shibuya lab), β-ACTIN (Sigma; A2228-100UL), MLH1 (BD Biosciences; 51-1327GR), γH2AX (EMD Millipore; 05-636), FLAG (Sigma; F1804-50UG), and MYC (MBL; M192-3); rat antibody against RPA2 (Cell signaling technology; 2208); sheep antibody against BRCA2 (Lee lab); and chicken antibody against SYCP3 (Shibuya lab). |
| Validation | The newly generated antibodies in this study were validated by western blotting as well as immunostaining using WT and knockout mouse controls. The other antibodies has been already validated in the published papers. |

## Eukaryotic cell lines

Policy information about cell lines

| | |
|---|---|
| Cell line source(s) | Mouse cell line: C2C12 (Sigma, Cat#91031101-1VL)<br>Mouse cell line: B16-F1 (Sigma, Cat#92101203-1VL)<br>Human U2OS DSB reporter cell line (Roger A Greenberg lab, pTUNER265) |
| Authentication | These cell lines are authenticated in the company (Sigma) and published papers (from Roger A Greenberg lab). |

| | |
|---|---|
| Mycoplasma contamination | We have confirmed that all cell lines was not contaminated with Mycoplasma. To further make sure to avoid contamination during the experiments, we added 2.5 µg/ml Plasmocin (InvivoGen) to the medium. |
| Commonly misidentified lines (See ICLAC register) | N/A |

## Animals and other organisms

Policy information about studies involving animals; ARRIVE guidelines recommended for reporting animal research

| | |
|---|---|
| Laboratory animals | We used WT and genetically modified mice (Spo11, Dmc1, Meiob, and Meilb2 KOs). Brme1 KO mice were generated in this study. All WT and knockout mice were congenic with the C57BL/6J background. We used adult (2 months old) male mice for most of the experiments, otherwise indicated in the figure legends. |
| Wild animals | No wild animal was used. |
| Field-collected samples | No wild animal was used. |
| Ethics oversight | All animal experiments were approved by the Institutional Animal Care and Use Committee (#1316/18). |

Note that full information on the approval of the study protocol must also be provided in the manuscript.
